# A narrative review of the effects of vitamin D3 on orthodontic tooth movement: Focus on molecular and cellular mechanisms

**DOI:** 10.1002/fsn3.4035

**Published:** 2024-02-16

**Authors:** Mohammad Yasin Zamanian, Maryam Golmohammadi, Filipp V. Vadiyan, Ausama A. Almulla, Diana E. Vadiyan, Natalia S. Morozova, Ola Kamal A. Alkadir, Anaheed Hussein Kareem, Mojtaba Alijani

**Affiliations:** ^1^ Department of Physiology, School of Medicine Hamadan University of Medical Sciences Hamadan Iran; ^2^ Department of Pharmacology and Toxicology, School of Pharmacy Hamadan University of Medical Sciences Hamadan Iran; ^3^ School of Medicine Shahid Beheshti University of Medical Sciences Tehran Iran; ^4^ Department of Therapeutic Dentistry, Institute of Dentistry I.M. Sechenov First Moscow State Medical University Moscow Russia; ^5^ Department of Dentistry Al Hadi University College Baghdad Iraq; ^6^ Department of Pediatric, Preventive Dentistry and Orthodontics, Institute of Dentistry I.M. Sechenov First Moscow State Medical University Moscow Russia; ^7^ Department of Medical Engineering Al‐Nisour University College Baghdad Iraq; ^8^ College of Health and Medical Technology Al‐Ayen University Thi‐Qar Iraq; ^9^ Department of Orthodontics, School of Dentistry Hamadan University of Medical Sciences Hamadan Iran

**Keywords:** bone remodeling, osteoclast, tooth movement, vitamin D3

## Abstract

Orthodontic tooth movement (OTM) is a critical process in dental alignment, driven by the application of calibrated orthodontic forces. This study delves into the intricate molecular and cellular mechanisms by which vitamin D3 influences OTM. Vitamin D3 is identified as a critical regulator in bone metabolism, enhancing osteoblast activity and bone formation while also modulating osteoclast quantity and RANKL expression, essential for the remodeling of the alveolar bone. The precise mechanisms through which vitamin D3 facilitates these processes are explored, highlighting its potential in accelerating bone remodeling and, consequently, tooth alignment. This comprehensive review underscores vitamin D3's anabolic impact on bone metabolism and its pivotal role in the synthesis and mineralization processes governed by osteoblasts. The findings illuminate vitamin D3's promise in augmenting orthodontic therapy, suggesting its utility in improving treatment efficiency and reducing duration. However, the need for further research into the optimal application of vitamin D3 in orthodontics is emphasized, particularly concerning dosage, timing, and delivery methods.

## Graphical abstract

Orthodontic tooth movement (OTM) is a complex biological process that involves bone remodeling. Vitamin D is known to play an important role in bone metabolism and can affect bone remodeling. Therefore, it is possible that Vitamin D could have some effect on OTM. Researchers found that patients with higher levels of Vitamin D had faster tooth movement compared to patients with lower levels of Vitamin D. The researchers suggested that Vitamin D supplementation could be a potential adjunctive therapy to enhance OTM in humans.

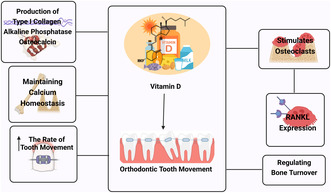

## 1 INTRODUCTION

Orthodontic tooth movement (OTM) is the process of moving teeth to correct misalignment and improve dental occlusion (Castroflorio et al., 2023). During OTM, mechanical forces are exerted on the teeth, remodeling the surrounding alveolar bone. This mechanism entails the formation of new bone on the tooth's tension aspect and the bone's resorption on the compression side (Yang et al., 2023). Orthodontists perform this procedure using braces or other orthodontic appliances (Ganesh & Pandian, 2017). However, OTM is not just a mechanical process; it also involves an inflammatory response and can cause tooth pain, also known as orthodontic pain (Zainal Ariffin et al., 2011). Additionally, OTM can result in changes in dental occlusion, which is the way the upper and lower teeth come together when biting or chewing (Wang et al., 2023). Individual responses to OTM differ markedly, with some patients acclimating smoothly to the process, whereas others might endure considerable discomfort or struggle with the adjustments in bite occlusion (Cioffi, 2023).

OTM is characterized by two principal mechanisms: bone resorption at the site of compressing force application and new bone formation where tensile forces are exerted. The coordination of these processes is regulated by a series of signaling components such as the receptor activator of nuclear factor kappa‐β ligand (RANKL), osteoprotegerin (OPG), and runt‐related transcription factor 2 (RUNX2). These elements are crucial in the metabolic activities of bone and periodontal tissues, which may be modified by introducing different biomaterials (Lin et al., 2023). Different biomaterials can affect the pace of OTM through various mechanisms of action (Farshidfar et al., 2022). Various hormones, ligands, and growth factors, such as prostaglandin E2 (PGE2), fibroblast growth factor (bFGF), and RANKL, have been studied for their potential to modulate OTM by affecting the cellular and molecular mechanisms involved in the process (Baleanu, 2021; Parcianello et al., 2022; Santana et al., 2020). PGE2, when injected locally, can accelerate OTM by bone resorption and promoting osteoclastogenesis (Seifi, Badiee, et al., 2013). Various methods and pharmacological agents are being explored to accelerate OTM. Some of these methods include vibration, piezo puncture, corticotomy, and laser therapy, as well as the use of pharmacological agents like corticosteroids and vitamin D3 (Ambashikar et al., 2021; Sarmah et al., 2011).

Vitamin D2 and vitamin D3 are both forms of vitamin D, but they differ in their sources and structures (Wilson et al., 2017). Vitamin D2, or ergocalciferol, is derived from plants and commonly found in fortified foods and supplements (Houghton & Vieth, 2006). However, vitamin D3, also known as cholecalciferol, is synthesized in the skin when exposed to sunlight and is also found in some animal‐based foods and supplements (Chandra et al., 2008). While the body utilizes both forms, vitamin D3 is believed to be more effective at raising and maintaining vitamin D levels than vitamin D2 (Heaney et al., 2011).

Vitamin D3 is biologically inactive and needs to undergo a series of hydroxylations in the liver and kidneys to become its active hormone, 1,25‐dihydroxyvitamin D3 (calcitriol). As the bioactive form of vitamin D, calcitriol is instrumental in the homeostasis of calcium and phosphorus—two minerals vital for the structural integrity of bones and teeth, as well as the maintenance of skeletal robustness (Adams & Hewison, 2010; Sosa Henríquez & de Tejada Romero, 2020). Beyond exposure to sunlight, dietary intake is another source of vitamin D3, with fatty fish like salmon and mackerel, cod liver oil, enriched dairy items, and dietary supplements all serving as contributors (Schmid & Walther, 2013). Adequate levels of vitamin D3 are essential for various bodily functions beyond bone health, including immune system function, muscle health, and cell growth regulation (Crescioli, 2022; Montenegro et al., 2019). Nevertheless, It is critical to recognize that an overabundance of vitamin D3 intake may lead to detrimental health effects, so it is recommended to maintain appropriate levels through a balanced diet and sensible sun exposure or under the guidance of a health‐care professional (Delrue & Speeckaert, 2023). Vitamin D3 toxicity can present with a range of symptoms, from gastrointestinal issues to neuropsychiatric and life‐threatening symptoms in severe cases. The classic symptoms of vitamin D3 toxicity are entirely attributable to hypercalcemia, and they include nausea, dehydration, and lethargy (Vieth, 2007). The function of vitamin D3 in bone metabolism is critical, and its implications for OTM have been the subject of research inquiries. Investigations using rat models have demonstrated that supplementation with vitamin D3 enhances bone morphometry, contributing to the stabilization of OTM (Gratton et al., 2022). An initial in vivo study assessed the potential of vitamin D3 to counteract the suppressive impact of sodium alendronate on OTM when administered systemically. Findings indicate that such co‐administration of vitamin D3 is capable of mitigating the repressive effects of sodium alendronate on OTM (Moradinejad et al., 2022).

Numerous research efforts have been directed toward understanding the effect of vitamin D3 on OTM, yet a comprehensive review article synthesizing these findings still needs to be included in the literature. This study seeks to clarify the impact of vitamin D3 on OTM by detailing the underlying cellular and molecular processes involved.

## 2 MECHANISM OF OTM

Mechanical forces applied to teeth elicit responses from both mineralized (such as the tooth structure and surrounding bone) and non‐mineralized tissues (such as the periodontal ligament and gingival tissues) (Krishnan & Davidovitch, 2009). Osteoclasts mediate bone resorption, while osteoblasts mediate bone formation (Zainal Ariffin et al., 2011). Comprehending the diverse biochemical pathways engaged in osteoclastogenesis and osteoblastogenesis offers the prospect of strategically influencing particular phases within these pathways to modulate orthodontic tooth movement. The OTM process is regulated by various cellular mechanisms, with osteocytes, and osteoblasts emerging as the principal mechanosensory cells. These cells sense mechanical forces and convert them into intracellular signals, producing various cytokines that orchestrate alveolar bone remodeling (Goulet et al., 2008).

The compression side is often viewed as the critical determinant in the pace of OTM, particularly concerning the aspect of bone resorption. Histological examinations have revealed that osteoclastogenesis begins on the compression side during the process of OTM (Cho et al., 2007; Iino et al., 2007; Mostafa et al., 2009; Wang et al., 2009). Interventions such as alveolar corticotomies, as well as non‐invasive techniques aimed at hastening tooth movement, are shown to substantially enhance the quantity and activity of osteoclasts (Cho et al., 2007; Collins & Sinclair, 1988; Kale et al., 2004; Kawasaki & Shimizu, 2000; Nishimura et al., 2008; Soma et al., 1999). Osteoclasts differentiate from multipotential hematopoietic precursors in the monocyte/macrophage lineage, and their genesis is contingent upon the action of mediators originating from stromal and osteoblastic lineage. This essential communication is mediated by the RANKL, which engages with its corresponding receptor RANK on osteoclastic progenitor cells (Goulet et al., 2008; Verborgt et al., 2002). RANKL is a pivotal protein synthesized by osteoblasts, the cells tasked with bone development (Low et al., 2005). It is instrumental in controlling osteoclastogenesis, the genesis of osteoclasts (Kobayashi et al., 2009). Osteoclasts are specialized cells that undertake bone resorption, which involves the breakdown and removal of aged or impaired bone tissue. The interplay between RANKL and RANK is vital for osteoclasts' differentiation, activity, and longevity (Xing et al., 2005). In contrast, OPG, originating from osteoblastic cells, functions as a competitive binding agent for RANKL, thereby obstructing the RANKL‐RANK interaction. This blockage by OPG effectively curtails osteoclastogenesis (Yasuda, 2021). Thus, the equilibrium between RANKL and OPG expression by osteoblastic cells, coupled with the expression of RANK on osteoclast precursors, is critical in the formation of active osteoclasts and the commencement of bone remodeling processes (Theoleyre et al., 2004). Numerous signaling entities and biomaterials that target the RANK/RANKL/OPG axis, including tumor necrosis factor α (TNF‐α), PGE2, and EGF, have been investigated to ascertain their effects on OTM (Cağlaroğlu & Erdem, 2012; Jin‐jie et al., 2018; Leiker et al., 1995; Seifi, Badiee, et al., 2013). An inflammatory cascade is initiated upon the administration of orthodontic pressure, marked by the increased synthesis and release of an array of cytokines. Within the first day, post‐application of orthodontic forces, human gingival crevicular fluid demonstrates a marked increase in interleukin‐1 (IL‐1), interleukin‐6 (IL‐6), and TNF‐α protein levels (Uematsu et al., 1996). Ren et al. (2007) observed a surge in the levels of TNF‐α, interleukin‐1β (IL‐1β), IL‐6, and interleukin‐8 (IL‐8) shortly following the initiation of tooth movement, particularly within a 24‐h timeframe. These Cytokines are noted for their role in enhancing osteoclast differentiation, functionality, and longevity. The upregulation of osteoclast activity is a pivotal element in triggering bone remodeling processes, which are essential for facilitating tooth movement (Glantschnig et al., 2003; Kayamori et al., 2010; Yao et al., 2008). Moreover, research indicates that mice deficient in TNF‐α expression exhibit a deceleration in tooth movement, underscoring the significance of this cytokine in the mechanics of OTM (Yoshimatsu et al., 2006). Prostaglandins are bioactive compounds produced by the body that play a role in various biochemical processes. Within this group, PGE2 acts as a potent modulator of bone metabolism. The external application of PGE2 has demonstrated efficacy in upregulating RANKL expression, a critical element in the development of osteoclasts. Numerous investigations carried out on rats using different doses of injected PGE2 have reported significant increases in the rate of OTM. Nevertheless, it is critical to acknowledge that these investigations have concurrently noted the adverse effect of root resorption (Cağlaroğlu & Erdem, 2012; Jin‐jie et al., 2018; Leiker et al., 1995). The naturally occurring polypeptide growth factor known as epidermal growth factor (EGF) is critical in bone metabolism and has been recognized for its role in tooth development and remodeling (Fisher & Lakshmanan, 1990). Expression of EGF can be identified within the dental follicle and alveolar bone during the pre‐eruptive phase of the tooth, indicating its participation in osteoclastic activities crucial for tooth eruption (Wise et al., 1992). Research has demonstrated that EGF can be utilized to enhance OTM. In investigative research, EGF was administered through submucosal injections close to the tooth where orthodontic force was applied (Alves et al., 2009; Saddi et al., 2008). When delivered in liposomes, EGF has been shown to promote bone resorption, increasing tooth movement (Saddi et al., 2008). In a separate study, EGF delivered in liposomes resulted in an upregulation of RANKL expression, as confirmed by the identification of osteoclasts using tartrate‐resistant acid phosphatase (TRAP) staining (Alves et al., 2009). A survey of existing literature has found that administering the nitric oxide (NO) precursor accelerates tooth movement in animal models. Conversely, the application of an NO synthase (NOS) inhibitor decelerates tooth movement (Yan et al., 2021). In separate research, Crawford et al. (2022) employed silica nanoparticles that release NO, which were injected directly into the area, to maintain the impact of NO on the movement of teeth over an extended period. Crawford's findings indicated that the release of NO from nanoparticles containing S‐nitrosothiols inhibited the movement of adjacent teeth for 1 week following the nanoparticle injection (Crawford et al., 2022). Although the results are promising, additional research is needed to fully understand NO's underlying mechanisms and clinical applications in OTM.

Besides bone tissue, the extracellular matrix (ECM) also responds to external mechanical forces and experiences remodeling (Li et al., 2008; Schwartz & DeSimone, 2008). The breakdown of collagen and additional macromolecules in ECM during force‐induced periodontal ligament (PDL) remodeling involves various enzymes, including serine proteases, aspartate proteases, matrix metalloproteinases, and cysteine proteases (Takahashi et al., 2003). Incorporating bioactive molecules into biomaterials, such as ethylene‐vinyl‐acetate, can alter the biochemical process of OTM by influencing the activities of enzymes involved in the process (Holliday et al., 2003). During OTM, non‐mineralized responses in the PDL include the reorganization and neovascularization of blood vessels and nerve fibers. Modulating these non‐mineralized responses can potentially impact osteogenesis and PDL remodeling, thereby influencing the overall paradental response to OTM (Krishnan & Davidovitch, 2006).

Additionally, the ECM undergoes remodeling in response to mechanical loads, involving enzymes such as matrix metalloproteinases (MMPs) that degrade collagen and other macromolecules (Li et al., 2008; Schwartz & DeSimone, 2008). In the dynamics of bone remodeling, alterations occur within the extracellular matrix. This involves decomposing and removing substances like collagen, coupled with the synthesis and deposition of new matrix components (Lin et al., 2023). MMPs, a family encompassing over 25 zinc‐dependent endopeptidases, are pivotal in regulating diverse biological mechanisms, including bone remodeling (Cui et al., 2017). Current research indicates a significant role for MMPs in activating osteoclasts amid bone resorption. A study by Holliday et al. revealed that the process of OTM could be attenuated by the localized administration of ilomastat, a broad‐spectrum MMP inhibitor, via ethylene‐vinyl‐acetate (ELVAX), a polymer characterized by its non‐biodegradable and non‐inflammatory properties, ensuring sustained release (Holliday et al., 2003). Mechanical forces applied during OTM have been found to enhance the expressions of vascular endothelial growth factor (VEGF) and its receptor VEGFR‐1 (Kaku et al., 2008; Kohno et al., 2014; Miyagawa et al., 2009; Nakai et al., 2009). A neutralizing antibody partially inhibits the induction of RANKL and VEGFR‐1 by mechanical stress to VEGF. This suggests that VEGF mediates RANKL induction through an autocrine pathway in response to mechanical force (Nakai et al., 2009).

**FIGURE 1:**
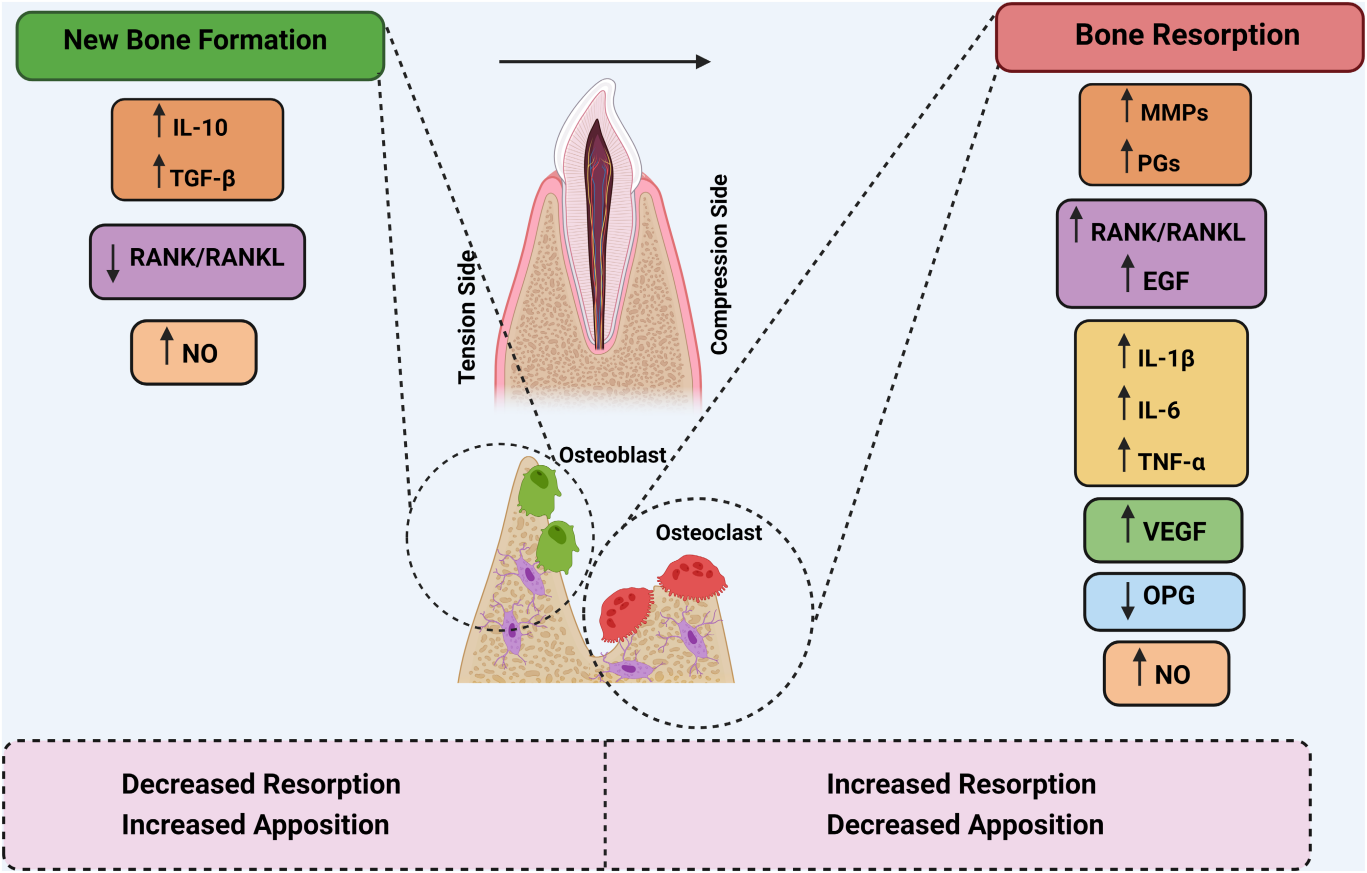
Schematic presentation of signaling factors related to compression and tension resulting from orthodontic loading.

Moreover, Street and Lenehan observed that VEGF has a potent anti‐apoptotic effect on cultured primary human osteoblasts. They also found that VEGF promotes the formation of nodules and enhances the differentiation of osteoblastic cells, as evidenced by increased expression of alkaline phosphatase and osteocalcin, which are marker molecules for osteoblast activity (Tan et al., 2010). Figure 1 summarizes the compression and tension signaling factors associated with orthodontic loading.

## 3 EFFECTS OF VITAMIN D3 ON OTM: EXPERIMENTAL STUDIES

Vitamin D3 positively affects healthy teeth (Botelho et al., 2020). It aids in absorbing calcium and phosphorus, essential for building and maintaining strong teeth (Hildebolt, 2005). It also helps form dentin, a vital component of teeth (Zhang et al., 2009). Adequate vitamin D3 levels can support oral health, reducing the risk of tooth decay and gum disease (Botelho et al., 2020). However, it is essential to maintain a balanced diet and good oral hygiene practices alongside sufficient vitamin D3 intake for optimal dental health (Swapna & Abdulsalam, 2022).

**FIGURE 2:**
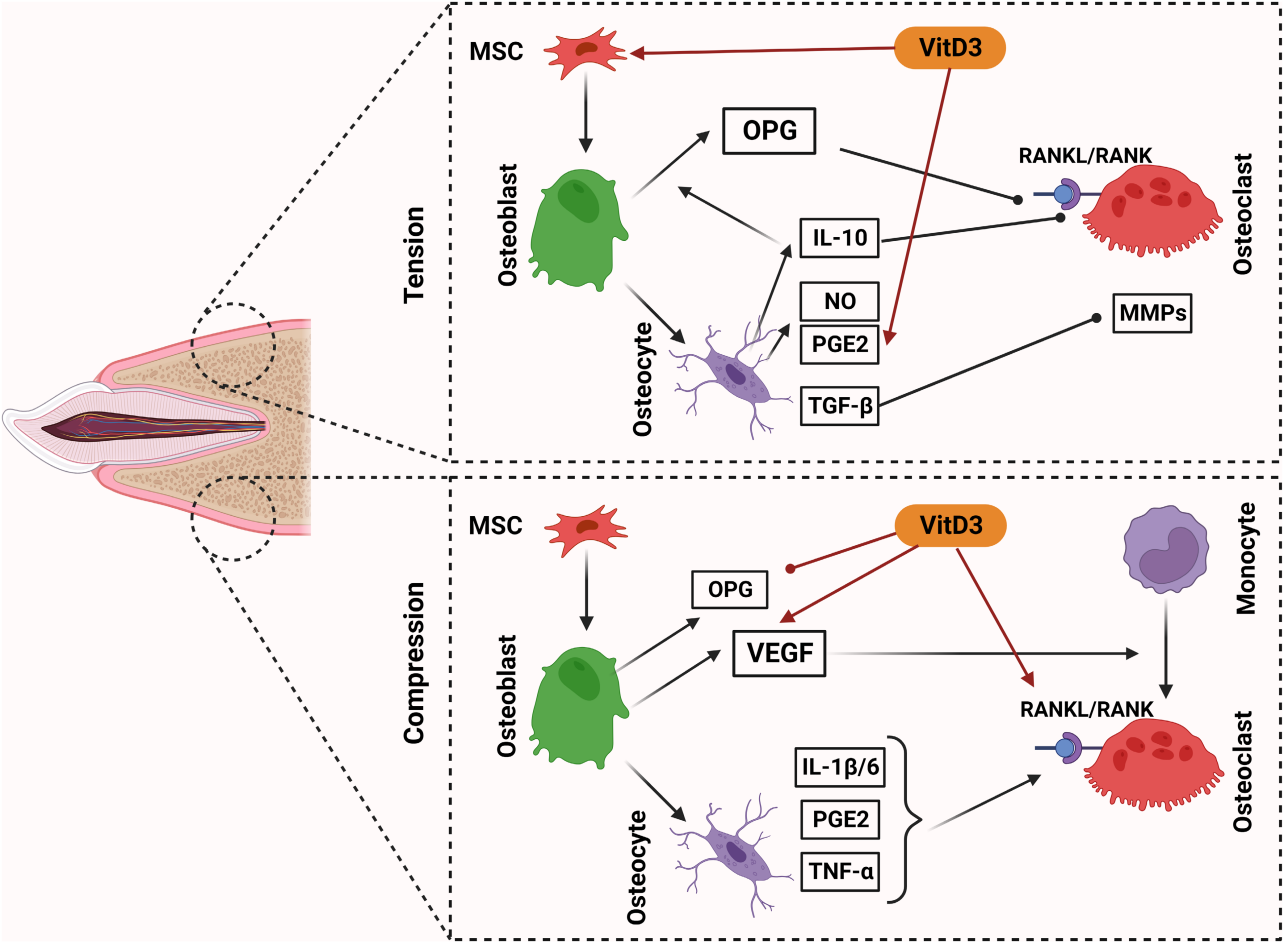
Vitamin D3 regulation effects during OTM at the compression and tension site.

Recent research has shed light on the multifaceted role of vitamin D3 in OTM, particularly emphasizing its regulatory effect on cellular functions crucial for this process. Emerging studies underline the significant impact of vitamin D3 in steering the cellular activities that drive the remodeling of alveolar bone, a critical phase in OTM. This underscores vitamin D3's integral role in enhancing bone adaptability and response during orthodontic treatments (Botelho et al., 2020; Indumathi & Sibyl, 2023; Moradinejad et al., 2022) (Figure 2).

The local application of vitamin D3 in orthodontic treatment has garnered attention due to its potential impact on accelerating OTM. Research conducted by Ciur et al. shed light on how localized vitamin D3 application could influence the dynamics of tooth movement. The results indicated that the treatment with vitamin D3 induced a dental movement that was, on average, 0.70 mm higher than control teeth. The dental movement was 1.70 mm for the test group and 1.00 mm for the control group, representing a 70% higher dental movement in the experimental group. Additionally, they found no significant statistical difference between the dental movement of men and women. The dental movement was observed to be more significant in the maxillary arch compared to the mandible, and the alignment duration did not seem to be associated with dental movement. They also revealed that the intra‐ligament administration of vitamin D3 increased the rate of tooth movement, and no root resorption was observed 3 months after the first administration of vitamin D3. These findings suggest that locally administered vitamin D3 may positively impact accelerating OTM without causing adverse effects on dental roots (Ciur et al., 2016).

Vascular endothelial growth factor (VEGF) proteins play a crucial role in angiogenesis, a process essential for tissue regeneration and healing in orthodontic treatments. The binding of VEGFs to their respective receptors, VEGFRs, triggers a cascade that enhances endothelial cell proliferation and motility, encourages the assembly of these cells into tubular structures, amplifies the permeability of blood vessels, and aids in sustaining the viability of vascular endothelial cells. These mechanisms are integral to angiogenesis (Bokhari & Hamar, 2023; Melincovici et al., 2018). The investigation by Nareswari et al. indicated that the administration of vitamin D was not associated with a substantial elevation in VEGF expression and the number of angiogenesis in OTM in pregnant rats. The lowest angiogenesis number was found in the control group at 7 days (C + 7), and the highest was found in the experimental group at 14 days (E+14). A notable discrepancy was observed between the C + 7 and E+14 groups and the E+7 and E+14 cohorts. Despite these differences, VEGF expression levels across the groups did not significantly differ. The study, therefore, infers that vitamin D3 administration does not markedly influence VEGF expression or angiogenic activity amidst OTM in pregnant rats (Nareswari et al., 2019).

Consideration of female hormonal fluctuations, especially during pregnancy, is essential in orthodontic treatment planning due to their potential impact on alveolar bone remodeling and OTM rates (Narmada et al., 2019). These hormonal fluctuations are known to influence alveolar bone remodeling during OTM. Female sex hormones substantially impact the oral milieu by engaging with receptors in the gingiva and affecting the function of fibroblasts and osteoblasts within the periodontal ligament (Jafri et al., 2015; Oliveira et al., 2013). Additionally, the practice of administering vitamin supplements like vitamin D3 to pregnant women aids in meeting the heightened nutritional needs that are advantageous for both the expectant mother and the developing child. Nevertheless, vitamin D3 is not exclusive to pregnant individuals; it also holds potential benefits for non‐pregnant individuals who may be facing insufficient levels or a deficit of this vitamin, affecting both genders (Soni et al., 2015).

Vitamin D3's role extends to stimulating osteoblast activity, a critical factor in stabilizing and enhancing the efficacy of OTM by promoting bone formation and remodeling (Sachan et al., 2013). A prior investigation disclosed that the intraligamentary injection of vitamin D3 in test subjects precipitated an appreciable 60% augmentation in the rate of OTM (Collins & Sinclair, 1988). Alkaline phosphatase of bone origin (BALP) is a pivotal enzyme in bone construction and mineralization (Nizet et al., 2020). Osteoblasts chiefly produce this enzyme (the architects of bone tissue). Recognized as an initial indicator of osteoblast maturation, BALP participates in creating bone matrix proteins (Przekora & Ginalska, 2015). A surge in BALP levels typically signals a heightened phase of bone synthesis, serving as a biomarker for the activity of bone remodeling (Kini & Nandeesh, 2012). Hisham et al. indicated that the post‐administration of vitamin D3 during OTM in pregnant rats did not significantly enhance BALP expression and osteoblast number. The highest number of osteoblasts occurred in the control group on day 7, and the highest BALP expression was in the experimental group on day 7. However, no statistically significant differences were observed in the expression of BALP between the groups. The study identified limitations such as the short observational period, small sample size, and limited molecular markers used. The conclusion emphasized the need for future long‐term studies with a higher sample size, better standardization of experimental procedures, and more markers for analysis to confirm the effects of vitamin D3 during OTM in pregnant rats (Hisham et al., 2019).

Kawakami, et al.'s research, elucidated that the localized delivery of vitamin D3 augments osteogenesis following experimental tooth displacement in rodent models. The histomorphometric evaluations revealed that on the seventh day, the absence of vitamin D3 correlated with diminished osteogenic rates (measured as mineral apposition rate, MAR) in regions undergoing compression. Contrastingly, rodents subjected to orthodontic intervention with concomitant vitamin D3 injections displayed a pronounced osteogenic response in the alveolar bone at the mesial aspect by day 14. This response was characterized by a significant rise in the MAR and an increase in the osteoblastic surface area on the tension side. These outcomes imply that the targeted application of vitamin D3 promotes the recovery of the supportive tissues, notably the alveolar bone, during post‐orthodontic procedures. The findings from this study advocate for the role of vitamin D3 in bolstering osteoblast‐driven bone regeneration within periodontal tissue, which is critical for dental stabilization after OTM (Kawakami & Takano‐Yamamoto, 2004).

PGE2 is a type of prostaglandin, a hormone‐like substance naturally produced in the body. PGE2 handles various physiological processes and acts as a potent multifunctional regulator of bone metabolism (Blackwell et al., 2010; Raisz, 1999). It is pivotal in inflammation, pain, and fever and regulates blood flow, smooth muscle contraction, and immune response (Narumiya & Furuyashiki, 2011). Seifi et al. showed that the combination of vitamin D3 and PGE2 had a synergistic effect on OTM in rats. The group that received both vitamin D3 and PGE2 had the highest amount of OTM compared to those that received only vitamin D3 or PGE2.

Additionally, the group receiving PGE2 had significantly faster OTM than the control group. However, the PGE2 group also significantly increased root resorption compared to the other groups. The group that received vitamin D3 showed significantly more tooth movement than the control group, but the amount of root resorption was similar to the control group. Overall, the combination of vitamin D3 and PGE2 helped to decrease root resorption and increase OTM (Seifi, Hamedi, et al., 2013). The limitations of PGE2 administration in orthodontic treatment include its potential to induce bone resorption, leading to increased root resorption. Exogenous PGE2 has been shown to increase the mRNA synthesis and protein secretion of the RANKL in osteoblasts, which can lead to osteoclastic activity and subsequent bone resorption (Mayahara et al., 2012). Additionally, PGE2 injections have been associated with a significant increase in root resorption compared to other treatment groups (Boekenoogen et al., 1996; Seifi, Hamedi, et al., 2013). Therefore, while PGE2 can accelerate orthodontic tooth movement, its administration may also lead to adverse effects such as root resorption, which should be carefully considered when using this treatment approach.

Sodium alendronate (ALN) is a medication classified as a bisphosphonate. It is commonly used for the treatment and prevention of osteolytic and osteopenic conditions such as osteoporosis (Wang et al., 2011). ALN works by reducing bone turnover, inhibiting the activity of osteoclasts (cells responsible for bone resorption), and promoting bone mineral density. It is available in various forms, including oral tablets and intravenous injections (Singh et al., 2015). Moradinejad et al. established that systemic delivery of vitamin D3 in rats treated with ALN could mitigate ALN's suppressive impact on OTM, restoring it to baseline conditions. Rats that received only ALN displayed reduced OTM measurements and diminished histological indications of bone remodeling compared to the control group.

Conversely, simultaneous administration of vitamin D3 with ALN resulted in OTM measurements and histological markers that were comparable to those of the control group. These findings suggest that systemic vitamin D3 could bolster OTM and elevate histological markers for bone metabolism. In contrast, ALN usage alone diminished OTM and its related histological biomarkers. Nonetheless, the combined administration of vitamin D3 with ALN appears to counterbalance the restrictive effects of ALN on OTM (Moradinejad et al., 2022).

Vitamin D3 administration during OTM in pregnant rats has been shown to affect RANKL expression and osteoclast number significantly. Narmada et al. found that administering vitamin D3 increased osteoclast number and RANKL expression, particularly in the group receiving vitamin D3 on day 7. This increase in osteoclast number and RANKL expression is consistent with previous research, demonstrating that vitamin D3 administration stimulates osteoclast activity, leading to bone resorption and faster tooth movement. Additionally, the study observed fluctuations in osteoclast number and RANKL expression, with a significant increase on day seven and a subsequent decline by day 14. These findings suggest that vitamin D3 can accelerate OTM by stimulating alveolar bone remodeling during pregnancy (Narmada et al., 2019). Further investigation is imperative to elucidate the detailed molecular pathways through which vitamin D3 influences osteoclast numbers and RANKL expression.

Relapse in orthodontics refers to the undesirable side effect where the teeth gradually return to their original position after orthodontic treatment (Martin, Littlewood et al., 2023). Studies have shown that a significant percentage of orthodontic patients experience relapse, with only a tiny proportion retaining acceptable alignment after an extended period (de Bernabé et al., 2017; Swidi et al., 2019; Yu et al., 2013). Various factors contribute to orthodontic relapse, including periodontal, muscular, anatomical, sex, occlusal, and orthodontic treatment‐related factors (Mohimd et al., 2018). The biological mechanisms of OTM and relapse are similar, involving tissue responses to initial force application, with the pressure side exhibiting increased osteoclast activity during OTM and a reduction in alveolar bone density, while relapse shows an increase in density (McManus et al., 2014).

Khamees and Al‐Groosh investigated the impact of vitamin D3 deficiency (VD3D) on OTM, retention, and relapse using a rat model. The results showed that VD3D led to a significant reduction in serum vitamin D3 levels and a slower rate of tooth movement. Additionally, VD3D was associated with a decrease in osteoblast cell count and total bone area and an increase in the relapse ratio. Conversely, the group that received systemic administration of vitamin D3 exhibited increased tooth movement, osteoblast cells, bone area, and reduced relapse ratio. The findings suggest that routine screening for vitamin D3 may be beneficial before commencing orthodontic treatment to optimize patient outcomes and reduce the risk of relapse (Khamees & Al Groosh, 2023). This study's vitamin D3 supplement group consisted of 11 rats that received two doses of 40,000 IU/kg of cholecalciferol via intramuscular injection on days 1 and 15 of the orthodontic treatment period.

In another study, Khamees et al. (2023) indicated that VD3D may delay the remodeling of the PDL, affecting the retention of teeth after orthodontic treatment. Conversely, vitamin D3 supplementation was associated with rapid PDL remodeling, potentially leading to increased bone healing and tooth stability after orthodontic retention. They found a significant difference in serum vitamin D3 levels at the end of orthodontic treatment, with the VD3D group showing the lowest levels. Additionally, histological evaluations revealed that the PDL borders appeared regular in the control and vitamin D3‐supplemented groups, while they appeared destructive in the VD3D group. They also observed a significant increase in PDL width in the vitamin D3‐supplemented group, followed by the control group, and the PDL width returned to normal more rapidly in these groups compared to the VD3D group. These findings suggest that vitamin D3 status is crucial in PDL remodeling and may affect orthodontic treatment outcomes and long‐term tooth stability (Khamees & Al Groosh, 2023). The vitamin D3 supplements were given at a dosage of 40,000 IU/kg of Cholecalciferol 300,000 I.U.\1 mL on days 1 and 15 of the orthodontic treatments.

We have summarized the results of some studies in Table 1.

**TABLE 1:** Experimental studies consisting of the purpose of the present review.

Authors	Dosage of vitamin D	Type of animal model	Results
Nareswari et al.	(0.2 mg/kg)	Rat	Vitamin D administration did not significantly increase VEGF expression and angiogenesis number in OTM of pregnant rats
Kawakami et al.	10^−10^M	Rat	The injection of 1,25(OH)_2_D_3_ led to increased osteoblast surface and mineral appositional rate, particularly on the mesial side of the interradicular septum
Moradinejad et al.	24,000 IU/kg	Rat	Systemic administration of vitamin D3 to rats receiving ALN could reverse the negative effect of ALN on OTM back to normal
Narmada et al.	(0.2 mg/kg)	Rat	Vitamin D supplementation increases the number of osteoclasts and RANKL expression during OTM in pregnant rats

## 4 EFFECTS OF VITAMIN D3 ON OTM: CLINICAL STUDIES

Vitamin D3 plays a significant role in tooth health by influencing bone biology and remodeling (Bartzela & Maltha, 2016). It regulates calcium absorption and the balance between osteoblasts and osteoclasts, which is essential for maintaining healthy bone structure, including the bones supporting the teeth (Anderson, 2017; Kawakami & Takano‐Yamamoto, 2004). Additionally, vitamin D3 produces type I collagen, alkaline phosphatase, and osteocalcin, which are crucial for bone formation and maintenance (Beresford et al., 1986; St‐Arnaud, 2008). Vitamin D3 helps mineralize teeth, which involves the formation of enamel and dentin. The ameloblast and odontoblast cells, responsible for forming enamel and dentin, respectively, have vitamin D3 receptors supporting the lowering of tooth decay risk (Al‐Jubori et al., 2022).

The vitamin D‐binding protein (VitDBP) serves a vital function in the systemic management and mobilization of vitamin D (Takahashi et al., 2003). It facilitates the binding and conveyance of various vitamin D metabolites, encompassing its precursor form and its activated variant, calcitriol (Galoppin et al., 2022). Tashkandi et al. conducted a pivotal study highlighting the correlation between salivary VitDBP levels and the OTM rate. The research revealed that maintaining VitDBP levels within the normal range significantly optimizes the rate of OTM, suggesting the potential of VitDBP as a biomarker for enhancing orthodontic treatment efficacy. The study's findings emphasize the nuanced relationship between vitamin D metabolism and orthodontic procedures, underscoring the importance of balanced VitDBP levels in achieving optimal tooth movement.

Additionally, they observed significant seasonal changes in VitDBP, with lower levels in the summer than in the winter. The research also identified a correlation between salivary VitDBP and alkaline phosphatase (ALP) levels during orthodontic tooth movement. Furthermore, the study highlighted the potential of salivary VitDBP as a measure of vitamin D3 metabolism, independent of age, race, and gender. The findings suggested the need for further research in larger cohorts to establish the predictive value of salivary VitDBP for tooth movement and to determine the impact of seasonal effects on VitDBP levels (Tashkandi et al., 2021).

Collins and Sinclair delved into the influence of vitamin D metabolites on OTM by administering local injections of 1,25‐dihydroxycholecalciferol. Their innovative approach revealed a significant enhancement in tooth movement, marking a 60% increase compared to the control group. This notable advancement underscores the potential of vitamin D in modulating bone remodeling and facilitating more efficient orthodontic treatments, thereby setting a foundation for future research in optimizing OTM strategies (Collins & Sinclair, 1988). This enhancement was attributed to the increased recruitment and activation of mononuclear osteoclasts, leading to greater alveolar bone resorption on the pressure side of the periodontal ligament. No apparent clinical, microscopic, or biochemical side effects were noted, indicating this approach's potential efficacy and safety in enhancing OTM (Collins & Sinclair, 1988). In summary, this study provides valuable insights into the potential use of vitamin D3 to enhance OTM, shedding light on the cellular mechanisms underlying this process and emphasizing the need for further research to explore its clinical applicability.

Platelet‐rich plasma (PRP), with its high concentration of platelets and essential growth factors, has been examined for its therapeutic potential in orthodontic treatments. PRP's unique composition fosters accelerated tissue regeneration and healing, vital for enhancing OTM and improving treatment outcomes (Davis et al., 2014; Perez et al., 2014). Platelets are small cell fragments in the blood that play a crucial role in clotting and wound healing (Nurden, 2018). When PRP is injected into a specific area, such as the site of an injury or during orthodontic treatment, the growth factors and proteins in the platelets can stimulate cell proliferation, angiogenesis, and tissue regeneration (Caruana et al., 2019; Kang et al., 2011; Li et al., 2014). In their investigation, Navya and colleagues discovered that the administration of PRP and vitamin D3 markedly hastened OTM when contrasted with their control groups. The duration of the study saw a consistently greater OTM rate in the PRP cohort than in the control.

However, this heightened OTM rate in the vitamin D3 group was primarily evident within the initial 2 months.

Moreover, the PRP group experienced a superior OTM rate relative to the vitamin D3 group. Both the PRP and vitamin D3 groups demonstrated reduced levels of root resorption in comparison to their controls. Of all teeth examined, the lateral incisors were most susceptible to root resorption, whereas the canines were least affected. The findings indicated PRP to be a more potent pharmacological mediator than vitamin D3 in promoting OTM (Navya et al., 2022).

Despite the promising results from preclinical and clinical studies, further research is still needed to fully understand the optimal dosage, timing, and duration of vitamin D3 administration for accelerating OTM. Additionally, the long‐term effects of vitamin D3 on tooth movement stability and root resorption need to be investigated. Nonetheless, using vitamin D3 as an adjunctive therapy in orthodontic treatment holds excellent potential for improving treatment outcomes and reducing treatment duration.

Al‐Attar and Abid investigated the effects of vitamin D3 on the alignment of mandibular anterior teeth, orthodontically induced root resorption (OIRR), and pain perception during the early phase of orthodontic therapy. The research was conducted as a multicenter, double‐blinded, randomized clinical trial involving adult patients aged 18–30 with moderate mandibular incisor crowding. The participants were divided into the normal vitamin D3 level group (ND3G) and the control group (CG). In the ND3G, the vitamin D3 level was measured and corrected to normal before starting orthodontic treatment, while in the CG, the vitamin D3 level was kept unknown until the completion of the alignment phase.

The study's results showed that having an optimal vitamin D3 level reduced the alignment time and pain associated with orthodontic treatment. The duration of lower incisor alignment therapy was shorter in the group with normalized vitamin D3 level, and the percentage of alignment improvement was significantly higher in this group during various stages of treatment. However, they found that vitamin D3 did not reduce orthodontically induced root resorption.

In addition, they reported a shorter time needed to align the mandibular incisors in the ND3G and a higher improvement percentage of the alignment at various time intervals. The research also supported the effect of vitamin D3 in increasing bone remodeling, thus accelerating OTM. Furthermore, they found that the average score of pain was less in the ND3G during the first 3 days of alignment compared to the CG during the same period, indicating a potential role of vitamin D3 in reducing pain associated with orthodontic treatment (Al‐Attar & Abid, 2022).

In summary, the study demonstrated that optimizing the level of vitamin D3 had positive effects on OTM, alignment time, and pain perception. However, it did not have a significant impact on reducing orthodontically induced root resorption.

## 5 CONCLUSION

This study accentuates the pivotal role of vitamin D3 in modulating the cellular and molecular dynamics critical to OTM. Vitamin D3 emerges as a key regulator in the orchestration of bone metabolism, influencing both the resorption and formation phases essential for effective tooth realignment. Specifically, it has been demonstrated to enhance the rate of OTM by modulating osteoblast and osteoclast activities, which are instrumental in alveolar bone remodeling. The cellular mechanism involves vitamin D3's interaction with osteoblast receptors, thereby driving the bone synthesis and mineralization processes crucial for OTM. Moreover, the upregulation of RANKL expression and the subsequent increase in osteoclast quantity highlight vitamin D3's potential in accelerating tooth movement through targeted bone remodeling. Collectively, these insights underscore the therapeutic potential of vitamin D3 in optimizing orthodontic interventions, offering prospects for more efficient treatment modalities. While the findings are promising, they underscore the necessity for further investigation into the precise dosage, timing, and application methods of vitamin D3 to harness its full potential in orthodontic therapy, ensuring optimized treatment outcomes and patient well‐being.

## AUTHOR CONTRIBUTIONS

**Mohammad Yasin Zamanian:** Conceptualization (equal); project administration (equal); supervision (equal); visualization (equal); writing – original draft (equal); writing – review and editing (equal). **Maryam Golmohammadi:** Project administration (equal); writing – original draft (equal); writing – review and editing (equal). **Filipp V. Vadiyan:** Methodology (equal); resources (equal). **Ausama A. Almulla:** Data curation (equal); resources (equal). **Diana E. Vadiyan:** Data curation (equal); resources (equal); validation (equal). **Natalia S. Morozova:** Methodology (equal); validation (equal). **Ola Kamal A. Alkadir:** Data curation (equal); methodology (equal); writing – original draft (equal). **Anaheed Hussein Kareem:** Data curation (equal); resources (equal). **Mojtaba Alijani:** Data curation (equal); writing – original draft (equal).

## FUNDING INFORMATION

Not applicable.

## CONFLICT OF INTEREST STATEMENT

The authors declare that they do not have any conflict of interest.

## INSTITUTIONAL REVIEW BOARD STATEMENT

Not applicable.

## INFORMED CONSENT STATEMENT

Not applicable.

## Data Availability

The data presented in this study are available in the article.
